# Prognostic value of miR-96 in patients with acute myeloid leukemia

**DOI:** 10.1186/1746-1596-9-76

**Published:** 2014-03-29

**Authors:** Jiangning Zhao, Quanyi Lu, Junfeng Zhu, Jianguo Fu, Yun-xian Chen

**Affiliations:** 1 Department of Hematology, the First Affiliated Hospital of Sun Yat-Sen University, 510080 Guangzhou, Guangdong, China; 2 Department of Hematology, Zhongshan Hospital of Xiamen University, 361004 Xiamen, Fujian, China; 3 Department of Pathology, the First Affiliated Hospital of Sun Yat-Sen University, 510080 Guangzhou, Guangdong, China; 4 Department of Nosocomial Infection Control Department, Zhongshan Hospital of Xiamen University, 361004 Xiamen, Fujian, China

**Keywords:** miR-96, Acute myeloid leukemia, Real-time quantitative RT-PCR assay, Prognosis

## Abstract

**Objective:**

Aberrant expression of miRNA (miR)-96 is associated with tumorigenesis and tumor progression in several solid cancers. However, little is known about the expression and prognostic value of miR-96 in acute myeloid leukemia (AML). Therefore, the aim of this study was to investigate the correlation of miR-96 expression with clinicopathological features and prognosis of AML.

**Methods:**

Real-time quantitative RT-PCR assay was performed to evaluate the expression levels of miR-96 in mononuclear cells from bone marrow or peripheral blood specimens in 86 patients with newly diagnosed AML.

**Results:**

Compared with normal controls, miR-96 expression was significantly downregulated in patients with newly diagnosed AML (*P* < 0.001). In analysis of 14 diagnosis/CR-paired samples, the expression level of miR-96 was found markedly elevated in patients after treatment than before (*P* < 0.001). Moreover, lower levels of miR-96 were associated with a higher white blood cell count, bone marrow blast count (*P* < 0.001 and 0.022, respectively), and lower hemoglobin and platelet count (*P* = 0.036 and 0.033, respectively). Although the low-expression group seemed to have a lower CR rate (53.85% vs 70.0%), there was no significant difference between the two groups (*P* = 0.213). The low-expression group had a lower relapse-free survival (RFS) (*P* = 0.038) and overall survival (OS) (*P* = 0.022) compared with the high-expression group during a median follow-up of 20 months.

**Conclusion:**

Our data demonstrated that the expression of miR-96 was downregulated in newly diagnosed AML patients and associated with leukemic burden, as well as RFS and OS. This suggests that miR-96 detection might become a potential biomarker of prognosis and monitoring in AML.

**Virtual slides:**

The virtual slide(s) for this article can be found here: http://www.diagnosticpathology.diagnomx.eu/vs/1434808553949498

## Introduction

Acute myeloid leukemia (AML) is a heterogeneous malignancy characterized by differentiation arrest and malignant proliferation of clonal myeloid precursors. Despite the fact that the majority of AML patients achieve complete remission (CR) after chemotherapy, only ~20% of patients achieve relatively long-term disease-free survival. Most of them die of either refractory or relapsed AML [1]. Karyotypes, many known valuable fusion genes in AML, and aberrant expression of several molecules, such as mutations in FLT3, C-Kit, NPM1, WT1, and CEBPA genes, are involved in diagnoses and prognosis of patients with AML. However, it is difficult to evaluate the survival and prognosis for most AML patients who lack characteristic cytogenetic or molecular changes. Thus, there is a need to find more valuable biomarkers to improve our understanding of the biology of leukemia. Recently, it was reported that variable miRNA expression signatures are also involved in the heterogeneity of AML [2-4].

miRNAs are a class of small noncoding single-strand RNA molecules that negatively regulate gene expression at the post-transcriptional level by binding to 3′-untranslated regions of their target mRNAs [5]. Increasing evidence supports a pivotal role for miRNAs in the multiple processes of carcinogenesis, including cell growth, apoptosis, differentiation, invasion, and tumor angiogenesis [6-10]. Recently, several studies indicated that expression of miRNAs is associated with patient survival, and they can function as prognostic and predictive indicators [11-14]. Moreover, it has been found that miRNA expression profiles are more accurate to classify tumors than mRNA profiles are [15], indicating that miRNAs could be used as molecular biomarkers for diagnosis of cancer and prediction of prognosis.

Several recent studies have demonstrated that the aberrant expression of miRNA (miR)-96 is associated with tumorigenesis and tumor progression in several types of cancer, including prostate cancer, urothelial carcinoma, bladder carcinoma, hepatoma, and breast cancer [16-20]. Little is known about the expression of miR-96 in AML, except for one study which used a microarray platform to scan for an miRNA signature [2]. However, no clinical correlation with miR-96 was further investigated. Thus, the aim of the present study was to investigate the correlation of miR-96 expression with clinicopathological features and prognosis of AML.

## Materials and methods

### Patients and tissue samples

From July 2010 to August 2012, 86 patients were diagnosed with *de novo* AML (non-M3) according to the French–American–British (FAB) criteria at Zhongshan Hospital of Xiamen University, China. The study was approved by the local ethics committee of Zhongshan Hospital of Xiamen University and informed consent was obtained from each patient or a family member. There were 42 male and 44 female patients, with a medium age of 46 (range 11–75) years. The median leukocyte count at diagnosis was 54,429/μL (range 710–315,000/μL). According to the FAB classification, three patients had AML M1, 32 had M2, 13 had M4, 34 had M5, three had M6, and one had M7. Sixty-five patients were subjected to cytogenetic classification according to karyotyping and detection of 16 types of common fusion genes, including AML1/ETO, PML/RARa, CBFβ/MYH11, MLL/AF10 and DEK/CAN and four types of mutations, namely, FLT3/ITD, NPM1, CEBPA and C-KIT. Clinical characteristics of the patients with AML are summarized in Table 1. Seventy-two patients received chemotherapy and were treated with standard cytarabine plus daunorubicin 7 + 3 induction chemotherapy. CR was defined by the criteria proposed by Cheson et al. [21]. Patients who achieved CR were then given high- or medium-dose cytarabine-based chemotherapy for consolidation according to their physical condition. Those who did not achieve CR were treated with medium-dose cytarabine-based chemotherapy. Forty-two patients with CR were followed up for a median 20 months (range 12–39 months). None of patients in CR had undergone stem cell transplantation until the end of follow-up because of the absence of suitable donors, poor condition, or other reasons. The data were censored when the patients relapsed or died. Bone marrow (BM) or peripheral blood (PB) specimens (peripheral white blood cells >50 × 10^9^) were obtained from patients at the time of their diagnosis, and from 14 patients in CR after two cycles of chemotherapy, after receiving informed consent. Five PB specimens from healthy donors for allogeneic hematopoietic stem cell transplantation were obtained as healthy controls, with informed consent.

**Table 1:** Clinical characteristics of 86 patients with newly diagnosed AML and expression of miR-96

**Characteristics**	**Cases**		**miRc96 expression level**		***x***^**2**^	**p**
			**Low expression**	**High expression**		
Sex	Male	42	28	14	0.374	0.541
	Female	44	32	12		
Age	<60	64	46	18	0.527	0.468
	≥60	22	14	8		
WBC	<10	26	11	15	13.323	0.000^*^
	≥10	60	49	11		
HGB	<80	54	42	12	4.415	0.036^*^
	≥80	32	18	14		
PLT	<50	48	38	10	4.550	0.033^*^
	≥50	38	22	16		
Blast in BM	<50%	37	21	16	5.212	0.022^*^
	≥50%	49	39	10		
FAB subtype	M1/M2	35	25	10	0.791	0.673
	M4/M5	47	33	14		
	Other	4	2	2		
Cytogenitics	Favorable	12	7	5	0.516	0.776
	Intermediate	39	26	13		
	Unfavorable	14	10	4		
Complete Remission	Y	42	28	14	1.551	0.213
	N	30	24	6		

*P<0.05.

### RNA extraction

Mononuclear cells from blood or BM specimens were isolated by Ficoll gradient (lymphoprep d = 1.077; Axis-Shield, Oslo, Norway) centrifugation for 20 min at 560 *g* at room temperature, and then washed and pelleted. Additionally, PB CD34^+^ cells from five healthy donors were enriched with immunomagnetic methods (Miltenyi Biotec, Bergisch-Gladbach, Germany; purity >90%). Total RNA was isolated using RNAprep Pure Blood Kit (Tiangen Biotech, Beijing, China), according to the manufacturer’s instructions, followed by treatment with RNase-free DNase I to remove contaminating genomic DNA. Finally, RNA was dissolved in RNase-free water. The RNA concentration was quantified by NanoDrop ND-1000 (Nanodrop, Wilmington, DE, USA) and stored at −80°C until use.

### RT-PCR

miR-96 and U6 snRNA-specific cDNA was formed using the Gene Amp System 9700 (Applied Biosystems, Foster City, CA, USA). U6 snRNA was used as an internal control. Reverse transcriptase primers for miR-96 and U6 were 5′-GTCGTATCCAGTGCGTGTCGTGGAGTCGGCAATTGCACTGGATACGACAGCAAAA-3′ and 5′-CGCTTCACGAATTTGCGTGTCAT-3′. Reverse transcription conditions contained 800 ng total RNA, 0.3 μl stem-loop RT primer (1 μM), 2 μl 10× RT buffer, 2 μl dNTP (2.5 mM each), 0.2 μl Moloney murine leukemia virus reverse transcriptase (Sangon, Beijing, China), and 0.3 μl RNase inhibitor (40 U/μl). The 20-μl reaction volumes were incubated at 16°C for 30 min, 42°C for 40 min, 85°C for 5 min, and then held at 4°C or stored at −20°C.

### Quantitative real-time PCR

The 10-μl PCR mixture included 2 μl cDNA, 5 μl 2× Master Mix (SuperArray Bioscience, Frederick, MD, USA), 0.5 μl primer-F (10 μM), 0.5 μl primer-R (10 μM), and 2 μl nuclease-free water. Forward and reverse primers for U6 (89 bp) were 5′-GCTTCGGCAGCACATATACTAAAAT-3′ and 5′-CGCTTCACGAATTTG-CGTGTCAT-3′. Primers for hsa-hsa-miR-96 (66 bp) were GSP: 5′-GGTTTGGCACTAGCACAT-3′; R: 5′-CAGTGCGTGTCGTGGAGT-3′. Real-time PCR was performed on an ABI PRISM7900 system (Applied Biosystems) with the following cycling conditions: 95°C for 10 min, followed by 40 cycles of 95°C for 10 s and 60°C for 1 min. The cycle threshold was automatically given by SDS 2.4 software (Applied Biosystems) and was defined as the fractional cycle number at which the fluorescence passed the fixed threshold of 0.15. The relative expression levels of miRNAs were calculated using the comparative ∆∆Ct method as described previously [22]. The fold changes in miRNAs were calculated using the 2(-Delta Delta C(T)) method [23]. All experiments were performed at least in triplicate.

### Statistical analysis

SPSS version 13.0 (IBM, Armonk, NY, USA) were used for statistical analysis. The Kruskal–Wallis nonparametric test was used to evaluate the significant difference of expression of miR-96 between the AML patients and NC. The *t* test was used to evaluate the difference of expression of miR-96 before and after chemotherapy. With regard to the correlation of leukemia clinical features with the expression level of miRNA-96, intergroup comparisons were performed using the χ^2^ test. Kaplan–Meier survival curves were used to determine any significant relationship between the expression level of miRNA-96 and the status of the patients with respect to relapse-free survival (RFS) and overall survival (OS). RFS was defined as the time between the achievement of complete remission and the time of the hematological relapse or the last follow-up. OS was defined as the time between the moment of diagnosis and death or the last follow-up. Differences were considered statistically significant when *P* was <0.05.

## Results

### Expression of miR-96 in AML patients

**Figure 1:**
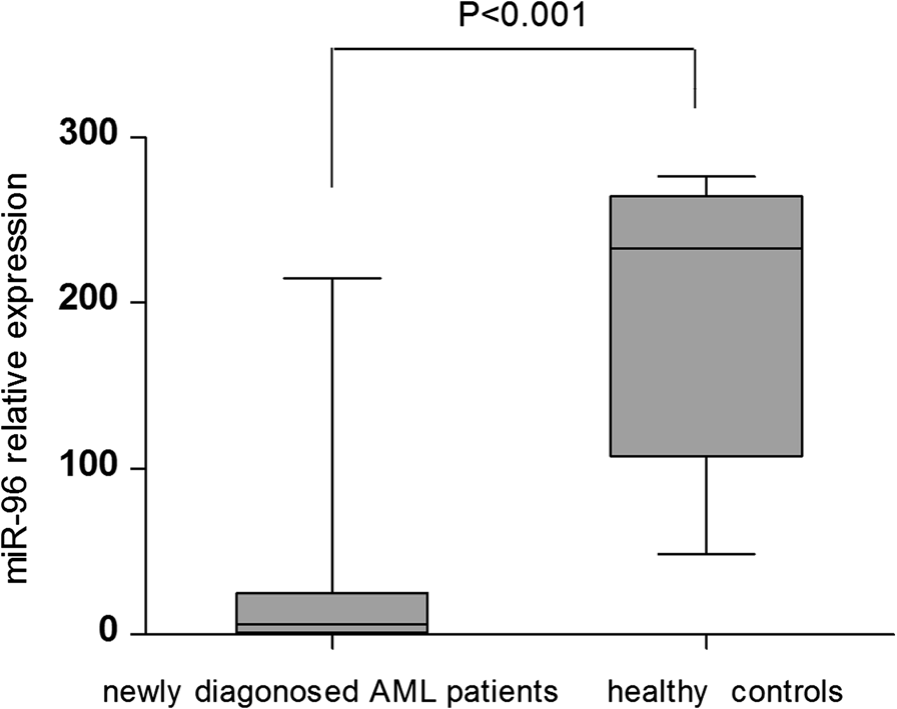
**Real-time PCR quantification of miR-96 expression in newly diagnosed AML patients and healthy controls.** miR-96 expression was significantly downregulated in AML patients when diagnosed (mean value 19.26) compared with those of healthy controls (mean value 195.43) (*P* < 0.001). U6 served as an internal normalized reference for miR-96. All experiments were performed at least in triplicate.

**Figure 2:**
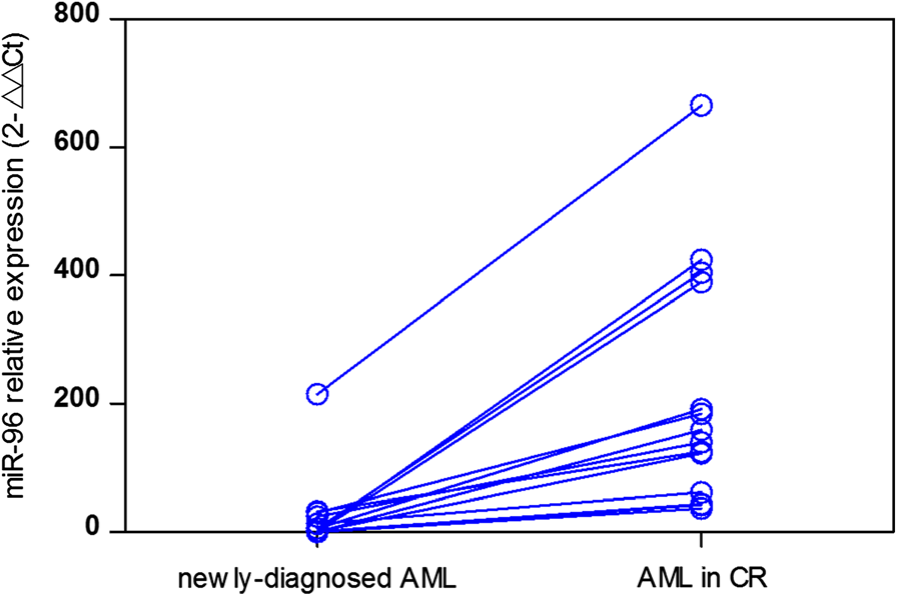
**Comparison of miR-96 expression before and after CR in 14 AML patients.** miR-96 level significantly increased after treatment (mean value 213.64) compared with before treatment (mean value 23.70, *P* < 0.001).

miR-96 expression was detected in BM/PB samples from patients with AML and normal controls. Expression of miRNA-96 was normalized with U6, and the values obtained were compared. Consequently, the relative abundances of miR-96 were significantly downregulated in AML patients (mean expression value 19.26, *n* = 86) compared with those of healthy controls (mean expression value 195.43, *n* = 5), which was a significant difference (Figure 1, *P* < 0.001). AML patients expressing miR-96 at levels less than the median (19.26) were assigned to the low-expression group (mean expression value 4.34, *n* = 60), and those samples with expression equal to or above the median value were assigned to the high-expression group (mean expression value 52.06, *n* = 26). Moreover, 14 AML patients who achieved CR after one or two cycles of chemotherapy were monitored for miR-96 during the course of treatment. The mean expression value of these AML patients when diagnosed was 23.70 and markedly increased to 213.64 when CR was achieved after chemotherapy (Figure 2, *P* < 0.001).

### Correlation of miR-96 expression with clinical characteristics of AML

The correlation of miR-96 expression with clinical characteristics at time of diagnosis is summarized in Table 1. Lower levels of miR-96 were associated with a higher white blood cell count, BM blast count (*P* < 0.001 and 0.022, respectively), and lower hemoglobin and platelet count (*P* = 0.036 and 0.033, respectively). Moreover, we failed to correlate the expression levels of miR-96 with other clinical parameters including sex (*P* = 0.541), age (*P* = 0.468), FAB subtype (*P* = 0.673) and cytogenetic abnormalities (*P* = 0.776).

### Association of miR-96 expression with clinical outcomes of AML

**Figure 3:**
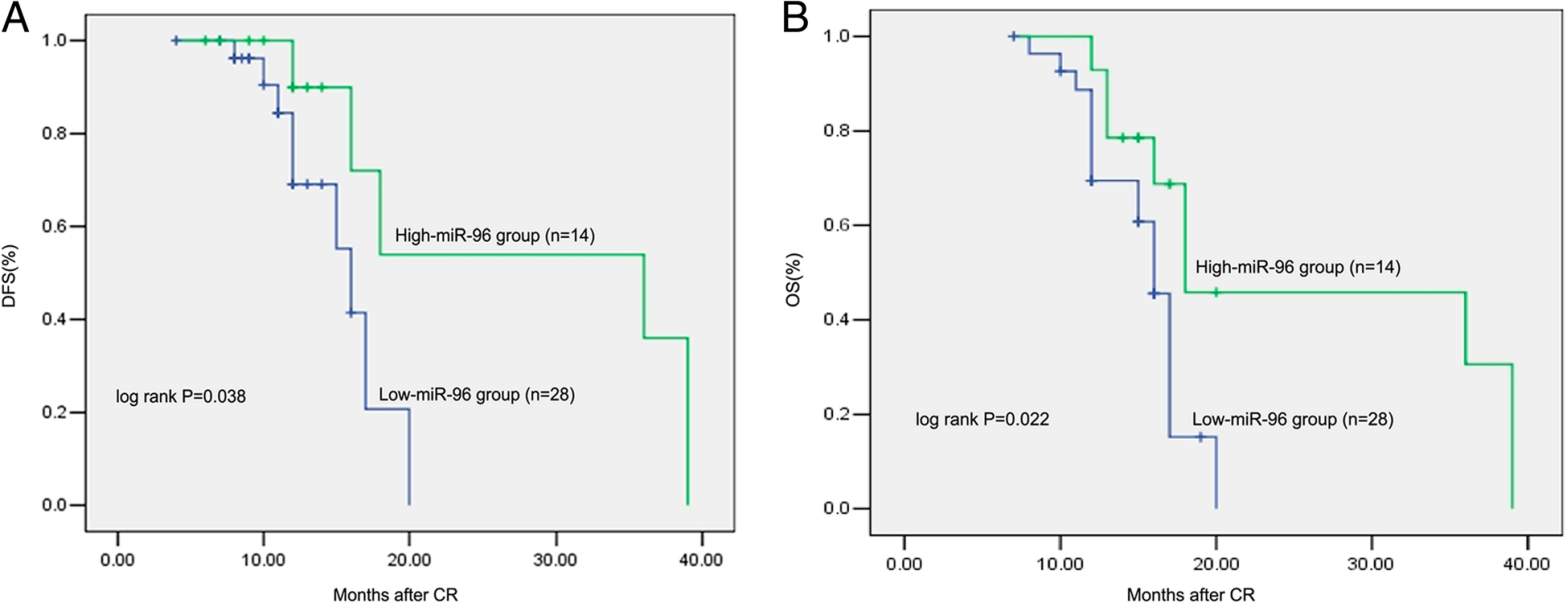
**Kaplan–Meier curves for RFS and OS stratified according to miR-96 expression in BM/PB samples from 42 AML patients in CR.** RFS(Figure 3**A**) and OS(Figure 3**B**) in patients with low miR-96 expression was significantly lower than that in those with high miR-96 expression (*P* < 0.05). The *P*-value was calculated using the log-rank test.

Seventy-two newly diagnosed patients received chemotherapy. The CR rate after two cycles of chemotherapy was 53.85% (28/52) in the low-expression group, compared with 70.0% (14/20) in the high-expression group. Although it seemed that the low-expression group had a lower CR rate, there was no significant difference between the two groups. Forty-two CR patients were followed up for a median duration of 20 months (range 12–39 months). The low-expression group had a shorter RFS (*P* = 0.038) and OS (*P* = 0.022). The Kaplan–Meier curves for PFS and OS stratified according to miR-96 expression in BM from AML patients in CR are shown in Figure 3.

## Discussion

Although more recurrent chromosomes, and even chromatin texture features [24], are relevant to AML, it is still not adequate for diagnosis and prognosis classification in most AML patients. Nowadays, considerable progress has been made in identifying, characterizing, and applying new molecular markers [25-27], including miRNAs. It is estimated that miRNAs may regulate up to one-third of the human genome. Thus they represent novel biological entities with potential value as tumor biomarkers, which can improve diagnosis, prognosis, and monitoring of treatment response for human cancers [14,28].

miR-96, together with miR-182 and miR-183, belongs to the miR-183-96-182 cluster, which has been demonstrated to play important roles in tumorigenesis and tumor progression [29-31]. Among these numbers, miR-96 may act as either an oncogenic miR-96 or play an antioncogenic role in many human malignancies. Several recent studies have demonstrated that aberrant expression of miR-96 is associated with tumorigenesis, tumor progression, and chemotherapy response in several types of cancer, including urothelial carcinoma, bladder carcinoma, hepatoma, glioma and breast cancer [17-20,30,32]. Overexpression of miR-96 has been observed in various types of cancer. In HepG2 hepatoma [19] and breast cancer [20] cells, overexpression of miR-96 induces cell proliferation and growth. Upregulation of miR-96 in breast cancer cells modulates their entry into the G1/S transitional phase, which is caused by downregulation of cyclin-dependent kinase (CDK) inhibitors, p27(Kip1) and p21(Cip1), and upregulation of the cell-cycle regulator cyclin D1 [20]. Moreover, miR-96 promotes the migration and invasion of hepatocellular carcinoma (HCCLM6) cells in vitro [32]. By contrast, accumulating studies have demonstrated that tumor-suppressive roles of miR-96 are found in other types of cancer. miR-96 levels are markedly decreased in pancreatic cancer [33]. In vitro and in vivo assays have established that miR-96 decreases cancer cell invasion and migration and slows tumor growth in a manner associated with KRAS gene downregulation. In ALK-expressing cancer cell lines and primary human tumors, transfection with miR-96 decreases levels of the different forms of ALK protein, and decreases the phosphorylation of ALK target proteins, resulting in reduced proliferation, colony formation, and migration [34]. However, the status of miR-96 expression and its prognostic roles are unclear in AML.

In this study, we detected the expression of miR-96 in BM from 86 patients with newly diagnosed AML, and showed that the relative expression level of miR-96 in AML was significantly lower than that in normal controls, which is consistent with the study of Garzon [2]. In that study, miR-96 was identified as one of 26 downregulated miRNAs in newly diagnosed AML, compared with mononuclear cells obtained from healthy donors. Then, we found that the level of miR-96 differed among the 14 diagnosis/CR-paired samples. The expression of miR-96 significantly increased after chemotherapy when patients achieved CR, suggesting that expression of miR-96 is consistent with tumor burden, and expression of miR-96 can be used as a prognostic marker of relapse. We analyzed the clinicopathological features of miR-96 in AML. Our results indicated that downregulation of miR-96 in AML was associated with a higher white blood cell count and bone marrow blast count, and lower hemoglobin and platelet count, which represented more aggressive clinical features and also related to a larger tumor burden. Finally, we analyzed the correlation of miR-96 expression with prognosis of AML patients, and found that patients with low miR-96 expression showed worse RFS and OS than those with high miR-96 expression, which implies that expression of miR-96 has an important value in AML prognosis classification.

## Conclusion

The results of our study indicate that expression of miR-96 was downregulated in patients with newly diagnosed AML and associated with leukemic burden, as well as RFS and OS. To the best of our knowledge, this is the first study to demonstrate that miR-96 was strongly correlated with the aggressive clinical features and poorer prognosis of AML, suggesting that miR-96 is a potential marker for risk stratification in the treatment of AML. However, there are many problems to be further investigated. For example, what is the mechanism by which miR-96 is downregulated in AML? Are there any correlations between the expression of miR-96 and some known prognosis factors, such as FLT3/ITD and NPM1 mutation? What is the prognostic value of miR-96 in the normal karyotype AML population when the population is further enlarged? This study is hypothesis generating, and further prospective analysis should be worth doing.

## Competing interests

The authors declare that they have no competing interests.

## Authors’ contributions

JNZ, QL and YC designed the study. JNZ carried out the experiments and drafted the manuscript; JFZ and JF participated in the experiments and data analysis. All authors read and approved the final manuscript.
